# Microglial activation mediates host neuronal survival induced by neural stem cells

**DOI:** 10.1111/jcmm.12281

**Published:** 2014-04-13

**Authors:** Hui-Mei Wu, Li-Feng Zhang, Pei-Shang Ding, Ya-Jing Liu, Xu Wu, Jiang-Ning Zhou

**Affiliations:** a CAS Key Laboratory of Brain Function and Diseases, School of Life Science, University of Science and Technology of ChinaHefei, Anhui, China; b Anhui Geriatric Institute, The First Affiliated Hospital of Anhui Medical UniversityHefei, Anhui, China

**Keywords:** neural stem cells, microglia, neuronal survival, Toll-like receptor, ERK1/2

## Abstract

The rational of neural stem cells (NSCs) in the therapy of neurological disease is either to replace dead neurons or to improve host neuronal survival, the latter of which has got less attention and the underlying mechanism is as yet little known. Using a transwell co-culture system, we reported that, in organotypic brain slice cultures, NSCs significantly improved host neuronal viability. Interestingly, this beneficial effect of NSCs was abrogated by a microglial inhibitor minocycline, while it was mimicked by a microglial agonist, Toll-like receptor 9 (TLR9) ligand CpG-ODN, which supports the pro-vital mediation by microglia on this NSCs-improved neuronal survival. Moreover, we showed that NSCs significantly induced host microglial movement and higher expression of a microglial marker IBA-1, the latter of which was positively correlated with TLR9 or extracellular-regulated protein kinases 1/2 (ERK1/2) activation. Real-time PCR revealed that NSCs inhibited the expression of pro-inflammatory molecules, but significantly increased the expression of molecules associated with a neuroprotective phenotype such as CX3CR1, triggering receptor expressed on myeloid cells-2 (TREM2) and insulin growth factor 1 (IGF-1). Similarly, in the microglia cells, NSCs induced the same microglial response as that in the slices. Further treatment with TLR9 ligand CpG-ODN, TLR9 inhibitor chloroquine (CQ) or ERK1/2 inhibitor U0126 demonstrated that TLR9-ERK1/2 pathway was involved in the NSCs-induced microglial activation. Collectively, this study indicated that NSCs improve host neuronal survival by switching microglia from a detrimental to a neuroprotective phenotype in adult mouse brain, and the microglial TLR9-ERK1/2 pathway seems to participate in this NSCs-mediated rescue action.

## Introduction

Neuronal loss is one of the hallmarks in most neurological disease [1]. The initial idea that neural stem cell (NSCs) transplants work *via* generating specialized cells to replace lost neurons [2,3] has been challenged, because it is considered to be less positive for the neurological disease in which massive neuronal loss occurs in large parts of the brain [4]. Recently, it has become recognized that grafted NSCs are capable of stimulating endogenous repair mechanisms and rescuing host neurons [5–8]. Cellular signalling may be one of the foundations of this co-ordinated actions and flexible responses [9,10]. However, a comprehensive understanding of the mechanisms by which stem cells cross-talk with the host nervous system is still lacking.

Microglia are the resident immunocompetent cells within the central nervous system (CNS) [11]. Upon activation, microglia produce a variety of effector molecules that have been closely associated with neurological disease [12,13], whereas they can also be involved in the maintenance of CNS homoeostasis by phagocytizing apoptotic bodies and cellular debris [14] through neuroprotective molecules [12]. An attractive approach to treat neurological diseases lies in the possibility of modifying the behaviour of microglia switching their functional phenotype from a detrimental to a protective one [13,15]. Microglia with neuroprotective features have been associated with an increased expression of the fractalkine receptor CX3CR1 [16], triggering receptor expressed on myeloid cells-2 (TREM2), insulin growth factor 1 (IGF-1) and to protect neurons by suppressing inflammatory gene expression [17,18]. Mesenchymal stem cells and neural stem/precursor cells are being extensively investigated for their ability to signal to the host microglia [19–21], and switch effector functions of cultured microglia.

Microglial functions and activity are conventionally considered to be modulated by a number of different stimuli *via* Toll-like receptor (TLR) [22] and p44/42 families of mitogen-activated protein kinase pathways (ERK1/2) [23–25]. Decreased activation of ERK1/2 was demonstrated in TLR-deficient microglia [26], suggesting that ERK1/2 is a key regulator of microglial activation induced by TLR [27,28]. In this respect, it is interesting to consider whether TLR or ERK1/2 signalling was involved in the cross-talk between NSCs and host microglia.

In the present study, we were prompted to study whether NSCs regulate resident microglial activity *via* TLR or ERK1/2 signal, and if this is true, whether this is responsible for the improved host neuronal viability.

## Materials and methods

### Animal

Adult ICR mice (8–10 weeks-old; Laboratory Animal Center, Shanghai, China) were housed under a 12-hr light–dark cycle (lights on 7:00 a.m.) at an ambient temperature of 24 ± 1°C. This study was approved by the Committee on the Ethics of Animal Experiments of the University of Science and Technology of China (Permit Number: USTCACUC0901001). All experiments were performed after mice were killed by ether inhalation, and all efforts were made to minimize suffering.

### BV2 microglia cultures

The murine microglia cell line BV2, was grown in DMEM (Invitrogen, Carlsbad, CA, USA) supplemented with 10% foetal bovine serum (FBS; Invitrogen).

### Primary microglia cells cultures

Primary microglia cells were isolated from 2-day-old ICR mice as described previously [29]. In brief, whole brains of neonatal mice were taken, blood vessel and meninges were carefully removed. Then, the whole brains of 12 mice were polled together and finely minced. Next, incubated with 0.25% trypsin-EDTA solution (0.25% trypsin and 1 mM EDTA; Invitrogen) at 37°C for 15 min. The enzymatic reaction was quenched by the addition of 20% FBS. After centrifugation at 200 × g for 10 min. at room temperature, the pellet was resuspended in the DMEM: Nutrient Mixture F-12 (Invitrogen) supplemented with 10% FBS. After 2 weeks, microglia cells were harvested by mild shaking of the flask and collected by centrifugation. Purity of microglia was analysed by IBA-1(1:3000; Wako, Osaka, Japan) cytohistochemistry.

### Preparation of mouse embryonic NSCs

**Fig. 1:**
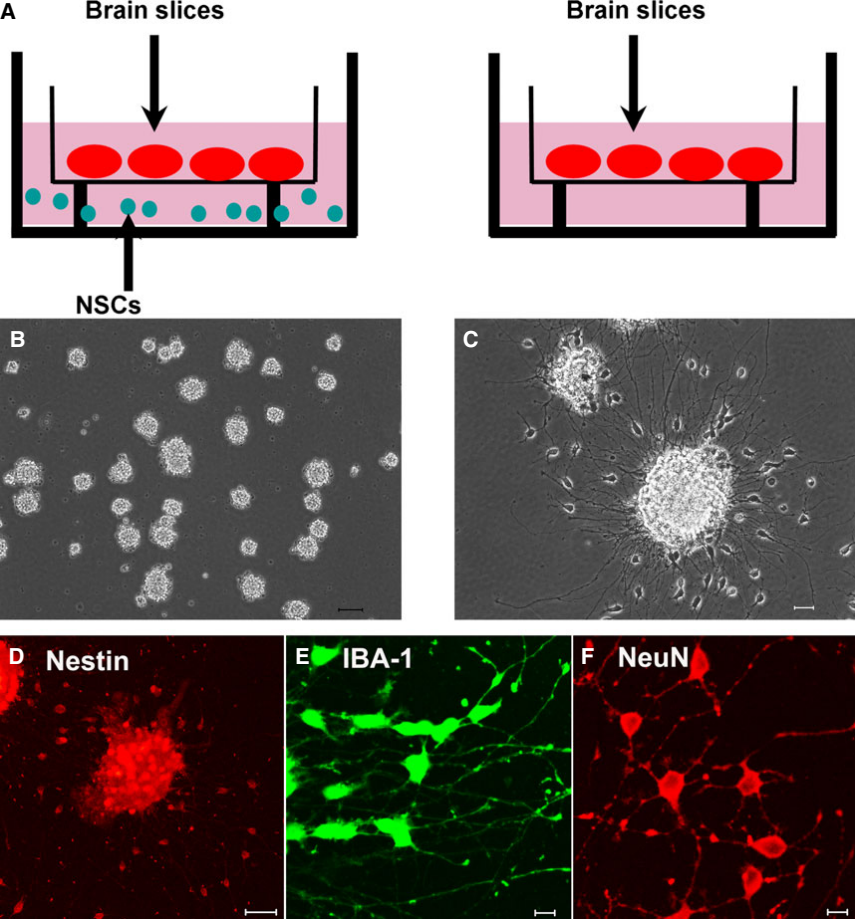
Co-cultured neural stem cells (NSCs) and the adult brain slices in a transwell system. (**A**) Co-culture the brain slices with or without NSCs separated by a membrane. (**B**) Representative images show 1-week-old neurospheres, bar: 50 μm. (**C**) Neurons derived by plating neurospheres and allowing them to grow without growth factors for 1 week, bar: 20 μm. (**D**) Cells migrating out of a neurosphere that had been allowed to attach to a substrate 7 days (nestin staining). After 7 days in serum-containing medium, many cells had differentiated towards several neural cell types (**E** and **F**), bar: 50 μm. (**E**) Cells expressing microglia maker IBA-1, bar: 10 μm. (**F**) Cells expressing neuron maker NeuN, bar: 10 μm.

Aged NSCs may provide an inhibitory environment for neuronal survival [30], whereas embryonic NSCs are reported to be capable of rescuing degenerating cells [6]. Thus, the embryonic NSCs were used in the present study. The embryonic NSCs were obtained by using a standard enzyme treatment protocol [31]. Briefly, timed pregnant ICR mice were killed by ether inhalation (E14, plug day = 0), then the foetuses were removed from the uterus and placed in ice-cold Hanks’ balanced salt solution (HBSS; Invitrogen). The forebrains were dissociated by using trypsin-EDTA solution (0.25% trypsin and 1 mM EDTA; Invitrogen), and the cells (∼10^5^ cells/ml) were plated in uncoated 6-well plates, with the DMEM: Nutrient Mixture F-12 (Invitrogen) supplemented with 2% B27 (Invitrogen), human recombinant basic fibroblast growth factor (b-FGF, 20 ng/ml; Pepro Tech, Rocky Hill, NJ, USA), human recombinant epidermal growth factor (EGF; 20 ng/ml; Pepro Tech), and heparin (5 μg/ml; Sigma-Aldrich, St. Louis, MO, USA). After 3–4 days *in vitro*, neurospheres were formed. After removing growth factors (EGF and FGF), the floating neurospheres (Fig. 1B) were allowed to attach to the bottom, bipolar-shaped nestin-positive cells started to migrate out of the spheres in serum containing medium (Fig. 1C and D). When these cells were allowed to stay attached for 1 week, they differentiated into different morphological shapes. The maker for microglia (IBA-1, Fig. 1E) and neurons (NeuN, Fig. 1F) were detected showing that E14 NSCs were multi-potent.

### Preparation of mouse brain slices

The brain slices were prepared as previously described [31,32]. When cultured *in vitro*, the cell viability in the brain slice is age-dependent. The cells in the neonatal or the young mouse (<10 days) brain slice survived well (Fig. S1A); however, the cells in aged mice brain slice (>12 months) died quickly (Fig. S1B). In the present study, male adult ICR mice (8- to 10-week old) were killed according to institutional guidelines, the brains were removed and placed in ice-cold HBSS (Invitrogen), and then sectioned the brains into 250 μm slices. The obtained slices were allocated systematically to 24-well plates to facilitate later sampling for co-culture experiments. The free-floating slices were cultured in neurobasal medium (NB, Invitrogen) supplemented with 2% B27 (Invitrogen) at 35°C in 5% CO_2_ for 24 hrs prior to co-culture with NSCs.

### Co-culture of adult brain slices and embryonic NSCs

The co-culture method we used was based on our previous study [31] as shown in Figure 1A. Briefly, Millicell inserts for organotypic culture applications (30 mm diameter with pore sizes of 0.4 μm; Millipore, Billerica, MA, USA) were used. Millicell inserts were standing in the uncoated 6-well plates, in a co-culture experiment, two wells were used: in each well, four slices were placed on the upper membrane of the insert. In the lower compartment of one well, we put harvested neurospheres (NSCs-treated group), while in the other well, no cells were added (mock-treated group; Fig. 1A and B). In the co-culture conditions, the medium was NB supplemented with 2% B27 without addition of any growth factors.

At the start of co-culture experiments, the neurospheres were harvested with centrifuge at 173 × g and rinsed with NB (Invitrogen) for three times to exclude the supplemented growth factors; no neurospheres older than 10 days were used for co-culturing. On average, 60 neurospheres were transferred into lower compartment of the co-culture well. Slices were transferred from the 24-well plates into each upper membrane of a millicell inserts in a systematic way such that they constituted comparable groups. The amount of NB (Invitrogen) was adjusted to 1.1 ml, which was sufficient to wet the exposed surfaces of the brain slices without submerging them. During the co-culture experiments, every other day 0.5 ml NB was exchanged and old neurospheres were replaced with newly harvested neurospheres.

### Co-culture of microglia cells and embryonic NSCs

Millicell inserts for cell culture applications (12 mm diameter with pore sizes of 0.4 μm; Millipore) were used. Millicell inserts were standing in the 24-well plates, BV2 cells were cultured in the 24-well plates. In a co-culture experiment, two wells were used: in each well, primary microglia cells or BV2 cells were cultured in the lower compartment. In the upper compartment of one well, we put 40 neurospheres harvested as mentioned above (NSCs-treated group), while in the other well, no cells were added (mock-treated group). The co-culture medium was NB supplemented with 2% B27 without addition of any growth factors.

### Viability assessment

Viability of cells in the slices was assessed by using the Live/Dead kit (Invitrogen) in accordance with the protocol provided by the manufacturer. The procedures for obtaining images and counting cells have been described previously [31]. Photographs at systematic randomly sampled locations on pyramidal layer III of the brain slices were obtained by using a CLSM (Carl Zeiss 510 inverted confocal laser scanning microscope, Thornwood, NY, USA) with a 20× objective. The work to distinguish the layers of the cultured slices was done according to the previous study [33]. Fifteen to thirty images were taken from each slice, and the cells were then counted by using 12 counting quadrants containing inclusion and exclusion lines. ‘Viable cells’ were defined as those that excluded ethidium bromide (because of their intact plasma membrane) and that contained a green fluorescent cytoplasm and a dark nucleus, whereas cells with a red nucleus only were counted as ‘dead cells’, and cells that had a red nucleus with green cytoplasm, and were consequently in an intermediate state between viable and dead, were denoted as ‘leaky cells’. The ‘total cell’ number was the sum number of viable cells, dead cells and leaky cells.

The number of cells per cubic micrometre was measured according to the previous work [33,34] with modification. Briefly, a counting frame containing 12 quadrants (60 × 60 μm) with exclusion and inclusion lines was superimposed over each image. The mean thickness of our counting volumes was ∼10.2 μm. Therefore, each counting block has a volume V = 60 × 60 × 10.2 cubic micrometre. Using the equation: cell density (/mm^3^) = cell number/(60 × 60 × 10.2 × 10^−9^) provides volumetric density estimates.

### Time-lapse imaging and generation of three-dimensional movies

The B4 isolectin from *Griffonia simplicifolia* seeds has been shown to be a useful marker of microglia in many CNS tissues [35]. A stock solution of Alexa Fluor-IB4 (200 μg/ml; Invitrogen) was prepared in water and stored frozen (−20°C). A final working solution (5 μg/ml) was prepared from the stock immediately prior to use. For fluorescence visualization of microglia, tissue slices were stained by using Alexa Fluor-IB4 as described elsewhere [36].

For time-lapse imaging, Alexa Fluor-IB4 stained slices were transferred to 35 mm glass-bottomed culture dishes (Mattek Corporation, Homer Ave. Ashland, MA, USA) and maintained on a heated microscope stage (35°C by forced warm air). In all cases, the imaging lasted 4 hrs as previously described [36]. To image cells in three dimensions within live slices, stacks of confocal optical sections were collected at 5 min. intervals. Such volume imaging provided a means of observing the three-dimensional structure and movements of live cells within the tissue volume. Each stack was composed of ∼10 optical sections (512 × 512 pixel arrays, two scans averaged per optical section) spanning up to 100 μm in the *z*-axis. For imaging relatively large tissue volumes and fields of view, a dry 20× objective was used. To generate three-dimensional movies of imaged tissue volumes, IMARIS 7.0 (Bitplane AG, Zurich, Switzerland) was used, and the images were exported as digital movies in MPG format. In the movies, microglia is classified as being immotile, motile or locomotory based on their motility behaviours [37]. Microglia whose cell bodies moved more than one cell-body diameter were classified as ‘locomotory’; microglia showing withdrawal or extension of branches were classified as ‘motile’; and microglia that did not move or change shape were classified as ‘immotile’.

### Quantitative RT-PCR

The brain slices or microglia cells were homogenized and total RNA was isolated by using Trizol Reagent (Invitrogen). Reverse transcriptase (Takara, Dalian, Japan) was used for cDNA synthesis. The PCR was performed by using the SYBR Green Kit (Takara) and a StepOne Real Time PCR System (Applied Biosystems, Foster City, CA, USA). The reaction volume was 25 μl, and the cycling parameters were 40 cycles of 15 sec. at 95°C and 60 sec. at 60°C. The sequences of the primer pairs (Table S1) were designed to span at least one intron to avoid amplification of DNA templates, and the relative amplification efficiencies of the primers were confirmed to be similar. The 2^−ΔΔCt^ method was used to calculate the relative amount of each target gene.

### Western blotting analysis

The brain slices or microglia cells were homogenized in radioimmunoprecipitation assay buffer (50 mM Tris-HCl, pH 7.4, 0.1% SDS, 1% NP-40, 0.25% sodium deoxycholate, 150 mM NaCl, 1 mM EDTA, 1 mM EGTA, and 1 mM Na_3_VO_4_). Prior to homogenization, a protease inhibitor cocktail (Roche, Indianapolis, IN, USA) and the phosphatase inhibitor PhosSTOP (Roche) were added. Proteins were separated on 12% SDS-PAGE gels and were then transferred to PVDF membranes (Millipore). Membranes were blocked in 5% bovine serum albumin (Sigma-Aldrich) and probed with antibodies against IBA-1 (1:400; Abcam, Boston, MA, USA), phospho-p44/42 MAPK (1:2000; Cell Signaling, Beverly, MA, USA), p44/42 MAPK (1:2000; Cell Signaling), TLR9 (1:500; Abcam) and tubulin (1:5000; Sigma-Aldrich). Bands were quantified by using Image J 1.38x software (NIH, Bethesda, MD, USA).

### Statistical analysis

Statistical analysis was performed by using SPSS for Windows, version 11.5. Data are expressed as the means ± SD. The differences in the number of viable, dead and leaky cells because of different treated times, the differences in the microglial morphological phenotype changes from the NSCs and the mock-treated group, the differences in the protein expression from mock-treated, NSCs, NSCs+CQ, NSCs+U0126, non-CpG-ODN, CpG-ODN, CpG-ODN+CQ and CpG-ODN+U0126 were analysed by using one-way anovas followed by post-hoc tests. The association between TLR9 and IBA-1, p-ERK1 and IBA-1 was examined by the Pearman's correlation coefficient. Other differences between groups were tested by using *t*-tests. Significant was set to *P* < 0.05.

## Results

### NSCs improved host neuronal viability in the adult brain slices by indirect contact

**Fig. 2:**
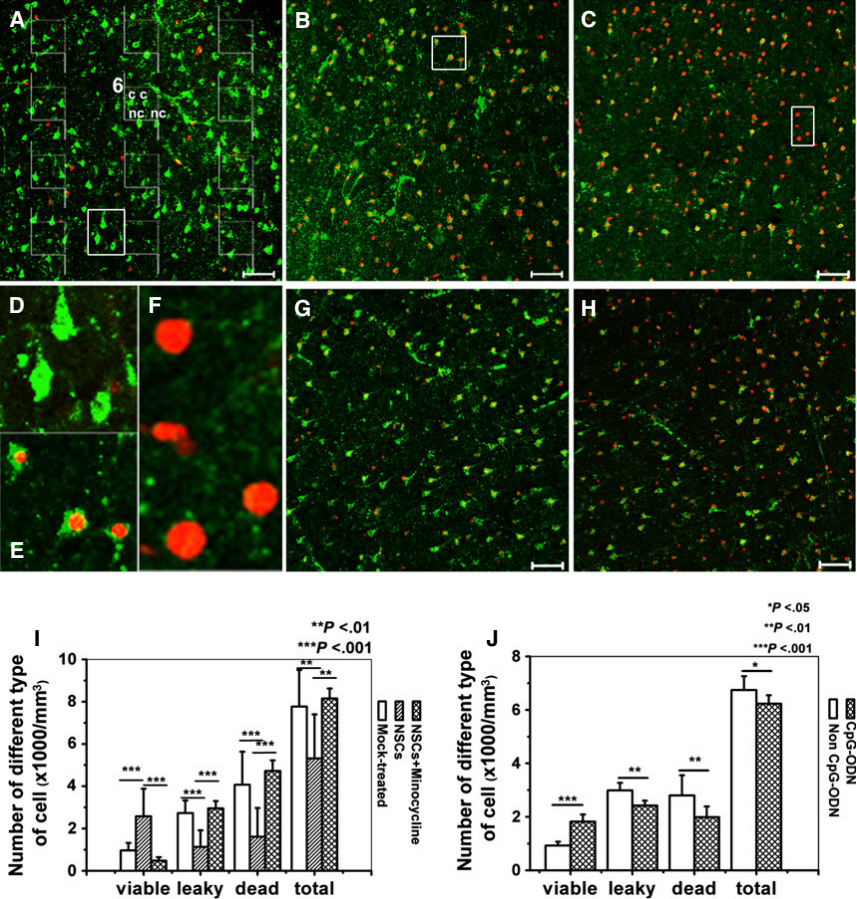
Representative images and quantification of different types of cells in adult mouse brain slices by using Live/Dead staining kit. The slices were stained by using Live/Dead kit as given in the Material and methods section. ‘Viable cells’ were defined as those that contained a green fluorescent cytoplasm and a dark nucleus, whereas cells with a red nucleus only were counted as ‘dead cells’, and cells that had a red nucleus with green cytoplasm, and were consequently in an intermediate state between viable and dead, were denoted as ‘leaky cells’. In each image, the cells were counted by using 12 counting quadrants containing inclusion and exclusion lines as shown in (**A**). Each counting field has an inclusion line (dashed) and an exclusion line (continuous). Any cell whose nucleus is in the frame without touching or crossing the exclusion line is counted. If a nucleus is touching or crossing the inclusion line, it is counted. For example, in field 6, two viable cells are counted (c), two viable cells are not counted (nc; if a cell profile is too vague, we choose to ignore it). (**A**) The slices co-cultured with neural stem cells (NSCs). (**B**) The mock-treated slices. (**C**) The minocycline-treated NSCs co-cultured slices. (**D**) The magnified image shows the viable cells outlined in (**A**). (**E**) The magnified image shows the leaky cells outlined in (**B**). (**F**) The magnified image shows the dead cells outlined in (**C**). (**G**) The CpG-ODN-treated slices. (**H**) The non-CpG-ODN-treated slices. (**I**) Quantification of different types of cells in the brain slices cultured for 3 days. (**J**) Quantification of different types of cell number in the brain slices treated with CpG-ODN or non-CpG-ODN. Results are shown as means ± SD (*n* = 16).

Co-culturing adult mouse brain slices with NSCs induced remarkable improvement in cell viability in the brain slices (Fig. 2A and B, Table 1). Neural stem cells co-cultured slices contained more viable cells, fewer leaky and dead cells compared with that in the mock-treated slices (Fig. 2I, Table 1). Surprisingly, the number of total cell was fewer in the NSCs co-cultured slices (*P* < 0.01; Fig. 2I, Table 1). According to our previous study, only cells with a pyramidal shape were counted as viable cells in both co-cultured slices and mock-treated slices, cells had a pyramidal shape indicating that they were neurons. Viability of small cells was more difficult to establish, because of the criterion of the presence of a dark nucleus. Leaky pyramidal neurons could be identified, but non-pyramidal-shaped leaky cell or dead cell could pertain to any cell type including pyramidal neurons, interneurons, glia and vascular cells. For this reason, the number of viable cells may have been underestimated.

**Table 1:** Comparison of the average cell numbers between slices co-cultured with neural stem cells and control slices on different cultured days

Group (±SD)	Treatment	Viable cells	Leaky cells	Dead cells	Total cells
DIV 3	NSCs	2.57 ± 1.31***	1.13 ± 0.79***	1.61 ± 1.36***	5.31 ± 2.08***
DIV 3	Mock-treated	0.97 ± 0.35	2.73 ± 0.60	4.07 ± 1.57	7.77 ± 1.73
DIV 7	NSCs	2.69 ± 0.67***	0.85 ± 0.24***	0.59 ± 0.24***	4.13 ± 0.67***
DIV 7	Mock-treated	0.80 ± 0.23	2.54 ± 0.56	3.91 ± 0.74	7.25 ± 0.51
DIV 10	NSCs	2.76 ± 0.78***	0.59 ± 0.34***	0.20 ± 0.32***	3.55 ± 1.31***
DIV 10	Mock-treated	0.51 ± 0.21	2.33 ± 0.99	4.43 ± 1.43	7.27 ± 1.15

NSCs: slices co-cultured with neural stem cells, Mock-treated: slices cultured without neural stem cells. ***P < 0.001 indicates significant difference between NSCs-treated and mock-treated groups at the same cultured days. The cell numbers are shown as per mm^3^ (×1000), DIV: day *in vitro*.

### Involvement of microglia in the NSCs-improved neuronal viability in the brain slices

To assess the possible role of the microglial activation in NSCs-improved neuronal viability, first, the effect of the microglial inhibitor on the neuronal viability was examined. Minocycline is widely used as an inhibitor of microglial activity [38,39], and we showed that minocycline had no effect on the multipotency and undifferentiated state of NSCs (Fig. S2). We treated the NSCs co-cultured brain slices with 10 μM minocycline for 3 days; the result showed that minocycline treatment significantly decreased the number of viable cells (*P* < 0.001), but increased the number of dead (*P* < 0.001) and leaky cells (*P* < 0.01; Fig. 2C and I).

Then TLR9 ligand CpG-ODN, a reported agonist of microglia was used and the result showed that after 3 days of treatment, CpG-ODN mimicked the effect of NSCs on the neuronal viability. 5 μM CpG-ODN 1668 (5′-TCC ATG ACG TTC CTG ATG CT-3′, Takara) significantly increased the number of viable cells, but decreased the number of dead and leaky cells (Fig. 2G and J) compared with that in the group treated with 5 μM non-CpG-ODN 1668(5′-TCC ATG AGC TTC CTG ATG CT-3′, Takara; Fig. 2H and J).

### NSCs cross-talked with resident microglia in the brain slices without direct contact

**Fig. 3:**
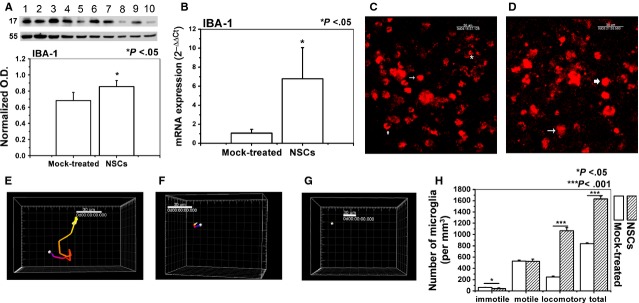
Neural stem cells (NSCs) induced the microglial activation in the brain slices. IBA-1 was used as a marker of activated microglia. (**A**) IBA-1 protein level in the slices. (lanes 1, 3, 5, 7 and 9, NSCs-treated group; lanes 2, 4, 6, 8 and 10, mock-treated group). (**B**) IBA-1 mRNA expression level in the slices. (**C**) Time-lapse images of microglia movement in the slices co-cultured with NSCs. (**D**) Time-lapse images of microglia movement in the slices from the mock-treated group. (**E**) The surpass image represents the cell (overstriking arrowed and arrowed in **C**), and the trajectory shows that it has large-scale movement. (**F**) The surpass image represents the cell (asterisk in **C** and overstriking arrowed in **D**), and the trajectory shows that the movement ability was limited. (**G**) The surpass image represents the cell (arrowed in **D**), and the trajectory shows its pseudopods keeping stretching out and retracting while the cell body does not move. (**H**) Quantitative analysis of microglia motility in the adult brain slices from the NSCs and the mock-treated group. Results are shown as means ± SD (*n* = 5).

To investigate the effect of NSCs on the microglial activation in the brain slices, the expression level of a microglial marker, ionized calcium binding adaptor molecule 1 (IBA-1), was checked by real-time quantitative PCR and western blotting; the morphological transformation of microglia was examined by visualizing the dynamic behaviour of microglia in the cultured brain slices. First, we found that IBA-1 expression was enhanced at both the protein (*P* < 0.05; Fig. 3A) and mRNA (*P* < 0.05; Fig. 3B) levels in the NSCs-treated slices compared with that in the mock-treated slices. To confirm that the effect of NSCs on the IBA-1 expression was not because of the different stimulation during slice preparation, we showed that before co-culturing with NSCs, the IBA-1 mRNA expression in the brain slices from the two comparable groups had no significant difference (Fig. S3A). Next, real-time living imaging of IB4-labelled microglia in live slices was performed to visualize the change in microglial movement; the number of microglia in the slices from the NSCs-treated group (Fig. 3C, Video S1) was more than that in the mock-treated group (*P* < 0.001; Fig. 3D, Video S2). Individual microglial morphology in time-lapse movies was categorized as locomotory (Fig. 3E, Video S3), motile (Fig. 3F, Video S4) or immotile (Fig. 3G, Video S5). Interestingly, there was an increase in the number of locomotory microglia, but a decrease in the number of immotile microglia in the NSCs-treated slices (*P* < 0.001, *P* < 0.05; Fig. 3H, Videos S1 and S2).

### NSCs affected the expression of inflammatory factors in the brain slices

**Fig. 4:**
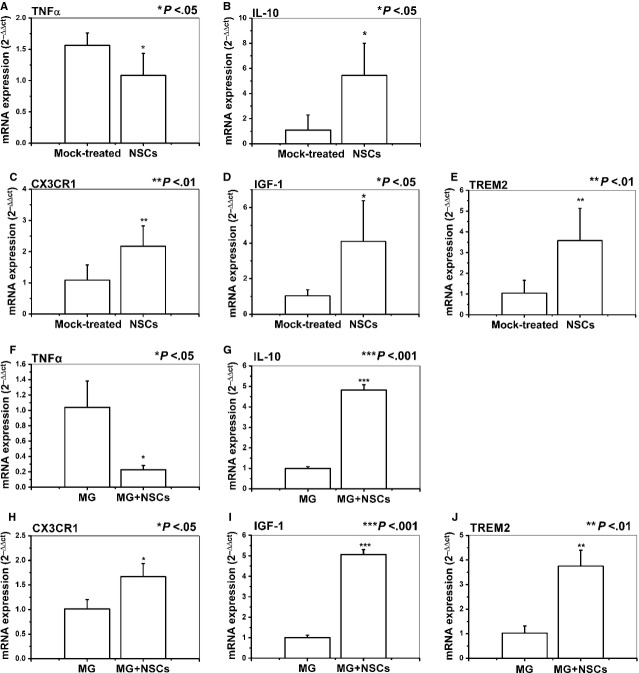
Neural stem cells (NSCs) affected the expression of effector molecules by microglia. The expression of effector molecules was analysed by real-time polymerase chain reaction as described in the Materials and methods section. (**A** and **B**) NSCs affected TNF-α and IL-10 mRNA expression in the brain slices. (**C**–**E**) NSCs increased CX3CR1, IGF-1 and TREM2 mRNA expression in the brain slices. Results are shown as means ± SD (*n* = 5). (**F** and **G**) NSCs affected TNF-α and IL-10 mRNA expression in primary microglia cells. (**H**–**J**) NSCs increased CX3CR1, IGF-1 and TREM2 mRNA expression in primary microglia cells. Results are shown as means ± SD of at least three independent experiments. Abbreviations: TREM2: triggering receptor expressed on myeloid cells 2; CX3CR1: CX3C chemokine receptor; IGF-1: insulin growth factor 1; TNF-α: tumour necrosis factor α.

To further assess the effect of NSCs on the microglial effector functions in the brain slices, we investigated the role of NSCs in the expression of different inflammatory factors produced by brain slices. We observed a significant decrease in TNF-α mRNA expression, whereas a significant increase in IL-10 mRNA expression when brain slices were co-cultured in the presence of NSCs (*P* < 0.05; Fig. 4A and B). However, before co-culturing with NSCs, the TNF-α and IL-10 mRNA expressions in the brain slices had no significant difference between the two comparable groups (Fig. S3B and C). Then, to assess whether results obtained from brain slices are representative of what could be observed in microglial cells, we co-cultured NSCs with the primary microglia cells or microglial cell line BV2 as described above and measured the inflammatory gene expression. Similar to that observed using brain slices in which microglia were representative of primary ones, NSCs significantly inhibited the expression of TNF-α and enhanced the expression of IL-10 in the primary microglia cells (*P* < 0.05, *P* < 0.01; Fig. 4F and G) or BV2 microglia cell line (*P* < 0.05, *P* < 0.01; Fig. S4A and B).

### NSCs increased the expression of neuroprotective molecules in the brain slices

Next, we examined whether NSCs could regulate the expression of molecules associated with microglial neuroprotective phenotype such as CX3CR1 and IGF-1 in the brain slices. The mRNA expression of CX3CR1 and IGF-1 was significantly up-regulated in NSCs co-cultured brain slices (*P* < 0.01, *P* < 0.05; Fig. 4C and D). Then, we addressed whether NSCs can affect the expression of TREM2, which was recently demonstrated to play a role in the maintenance of CNS homoeostasis without inflammation [40]. Similarly, we detected a significant up-regulation of TREM2 expression in NSCs co-cultured brain slices (*P* < 0.01; Fig. 4E). In line with that observed by using brain slices in which microglia are representative of primary ones, NSCs also up-regulated the expression of CX3CR1, IGF-1 and TREM2 in the primary microglia cells (*P* < 0.05, *P* < 0.01, *P* < 0.001; Fig. 4H–J) or BV2 cells (*P* < 0.05; Fig. S4C–E). It was supposed to assess these genes expression described in brain slices directly on primary microglia cells; however, because of the low-yield of primary microglia cells, to obtain enough total RNA for gene expression studies (>5 μg of total RNA), it is necessary to pool cells from 6 to 8 mice (brains/spinal cords) [41]. The total cell number in the brain slices was much less than the requested number of cells for the isolation and the subsequent gene expression assessing. However, the similarity of the results obtained utilizing brain slices in which microglia are representative of primary ones and the primary microglia cells or BV2 cell line suggests that the latter is a robust and reproducible tool to evaluate the effect of NSCs on microglia. Therefore, the latter experiments were carried out on primary microglia cells or BV2 cells.

### Involvement of TLR9 and ERK1/2 in the NSCs-induced microglial activation

**Fig. 5:**
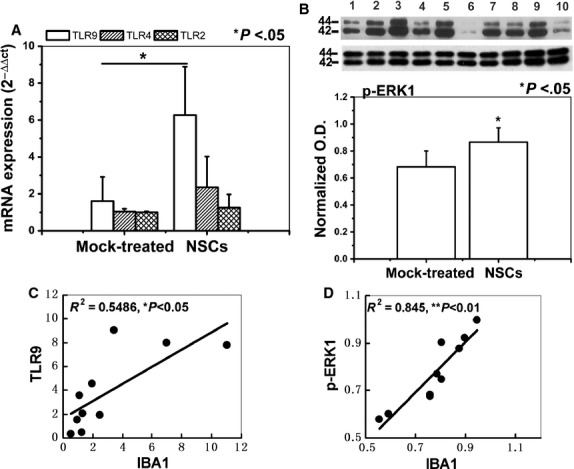
Neural stem cells (NSCs) induced microglial activation *via* TLR9 and ERK1/2. (**A**) The real-time PCR analysis of the TLR9, TLR2 and TLR4 mRNA expression level in the slices co-cultured with or without NSCs. (**B**) p-ERK1 phosphorylation level in the slices. (lanes 1, 3, 5, 7 and 9, NSCs-treated group; lanes 2, 4, 6, 8 and 10, mock-treated group). (**C**) TLR9 mRNA level positively correlated with IBA-1 mRNA level. (**D**) p-ERK1 protein level positively correlated with IBA-1 protein level. Results are shown as means ± SD (*n* = 5).

To further investigate the possible underlying mechanism by which NSCs regulate microglial activity, we addressed the possible effect of NSCs on the expression of TLR and phosphorylation of the p44/42 MAPK, which have been reported to be involved in the microglial activation. Real-time quantitative PCR revealed that TLR9 mRNA level, but not TLR4, TLR2, was significantly higher in the NSCs co-cultured brain slices compared with that in the mock-treated group (*P* < 0.05; Fig. 5A). Western analyses indicated that the ERK1 phosphorylation level was increased significantly in the NSCs co-cultured group (*P* < 0.05; Fig. 5B). Interestingly, a significant correlation was found between TLR9 and IBA-1(pearson *r*^2^ = 0.5486, *P < 0.05, Fig. 5C), p-ERK1 and IBA-1 (pearson *r*^2^ = 0.845, **P < 0.01, Fig. 5D) respectively. These results indicated that NSCs-induced microglial activation may be mediated by the TLR9 and ERK1/2 signal.

### NSCs induced microglial activation *via* TLR9-ERK1/2 pathway

**Fig. 6:**
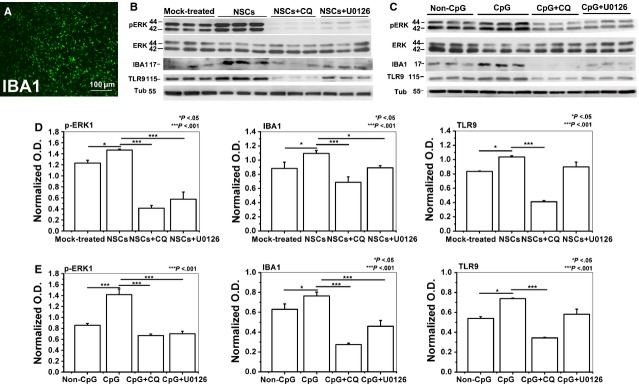
Microglia was activated *via* TLR9-ERK1/2 pathway in primary microglia cells. (**A**) Representative image of isolated primary microglia cells stained by IBA-1 antibody, bar: 100 μm. (**B**) Western blotting analysis shows the protein expression of ERK1/2, TLR9 and IBA-1 in primary microglia cells after 3 days of co-culture with or without neural stem cells (NSCs), or with NSCs in the presence of CQ, U0126. (**C**) Western blotting analysis shows the protein expression of ERK1/2, TLR9 and IBA-1 in primary microglia cells after 24 hrs treated with non-CpG, CpG, CpG+CQ, CpG+U0126. (**D**) Data show the protein level of TLR9, phosphorylated ERK1 and IBA-1 in primary microglia cells co-cultured with or without NSCs, or with NSCs in the presence of CQ, U0126. (**E**) Data show the protein level of TLR9, phosphorylated ERK1 and IBA-1 in primary microglia cells treated with non-CpG, CpG, CpG+CQ, CpG+U0126. Data are expressed as means ± SD from at least three independent experiments.

To verify whether NSCs-induced microglial activation was *via* TLR9 and ERK1/2, primary microglia cells and NSCs were co-cultured separated by a membrane; the results displayed that 3 days of NSCs co-culturing increased TLR9 and IBA-1 protein expression, and enhanced ERK1 phosphorylation of BV2 cells (Fig. 6B and D), whereas 5 μM chloroquine treatment abrogated this effect of NSCs, 10 μM U0126 treatment decreased NSCs-induced ERK1/2 activation and IBA-1 expression, but not the TLR9 expression (Fig. 6B and D). Furthermore, incubation with 5 μM TLR9 ligand CpG-ODN1668 for 24 hrs significantly increased the protein expression of TLR9, phosphorylated ERK1 and IBA-1 in primary microglia cells (Fig. 6C and E). CpG-ODN1668 and 5 μM chloroquine simultaneous treatment decreased CpG-ODN1668-induced ERK1/2 activation, IBA-1 and TLR9 expression, whereas, CpG-ODN1668 and 10 μM U0126 simultaneous treatment decreased CpG-ODN1668-induced ERK1/2 activation and IBA-1 expression, but not the TLR9 expression (Fig. 6C and E). Consistent with that observed in primary microglia cells, we demonstrated that in the BV2 microglia cell line, chloroquine treatment abrogated the effect of NSCs or CpG-ODN on the protein expression of TLR9, IBA-1 and phosphorylated ERK1, whereas U0126 treatment decreased NSCs- or CpG-ODN-induced ERK1/2 activation and IBA-1 expression, but not the TLR9 expression (Fig. S5). Thus, we drew a conclusion that microglia may be activated *via* TLR9-ERK1/2 pathway.

## Discussion

We demonstrated here that culturing adult brain slices separated by a membrane from NSCs resulted in robust improvement of host neuronal viability. Interestingly, this effect was abolished by a microglial inhibitor, but mimicked by a microglial agonist. Moreover, NSCs displayed a remarkable ability to modulate several features of microglia. Neural stem cell co-culturing was capable of inhibiting the expression of pro-inflammatory factor, but increasing the expression of anti-inflammatory factor, CX3CR1, TREM2 and IGF-1, which are associated with microglial neuroprotective phenotype. We hypothesize that NSCs may switch microglial functional phenotype from a detrimental to a protective one, and this may contribute to the improved neuronal viability in the brain slices. Furthermore, TLR9-ERK1/2 pathway may play an important role in this NSCs-induced microglial activation.

Grafted NSCs can protect or rescue endogenous neurons rather than to replace lost ones, as first shown after spinal cord injury and established in neurodegenerative conditions in mice [7] and monkeys [42]. Here, we found that NSCs enhanced the density of viable cells in adult mouse brain slices by indirect contact, a result similar to that found in human brain slices [31]. This improvement occurred within 3 days after start of the treatment, but stayed at roughly the same level after longer treatment. According to the counting method mentioned above, although non-pyramidal-shaped leaky or dead small neurons were already included in the cell number analysis, small cells such as some GABAergic neurons as viable cells may be missed. For this reason, the number of viable cells may have been underestimated, thus this counting method strengthened the conclusion that NSCs improved the neuronal survival in the brain slices. Simultaneously, NSCs treatment significantly decreased the mean number of dead cells and total cells over time (data showed in Fig. S6B and D), suggesting that part of the missing cells may have been degraded or cleared during co-culturing period.

Microglia are the resident macrophages of the CNS [11], and belong to the innate arms of immune system. The alteration of their morphology and phenotype is necessary for dead cells removal [43,44]. Scientific advancements during the last few years have shown that immune-related factors impair neuronal survival and induce neuronal death. Therefore, immunomodulation should benefit stem-cell therapy [10]. In this study, a microglial inhibitor, minocycline treatment abolished the neurorescuing effects of NSCs, accompanied by failing to decrease the dead and leaky cells number. Minocycline has been shown to have neuroprotective effects by attenuating microglial activation in adult global and focal ischaemia models [45,46]. However, there are lines of evidence suggesting that minocycline may also have detrimental effects (severe neuronal loss) in some animal models of neurological disease, because of the inhibition of repair mechanisms mediated by microglia [47,48]. The contradictory results may be attributable to the effect of minocycline on different microglial phenotype [49]. Microglia can act detrimentally or beneficially for the surrounding cells [50]. The beneficial microglia phenotype is thought to be associated with phagocytosis, which contributes for neurons survival [51–53], whereas the detrimental microglia phenotype is considered to release cytotoxic factors [54]. In the present study, NSCs might instruct microglia towards a beneficial phenotype, whereas minocycline reversed this effect.

On the other hand, a microglial agonist, TLR9 ligand CpG-ODN, mimicked the effect of NSCs and improved the neuronal viability. Although it is reported that stimulation of microglia *via* TLR9 was associated with neuronal injury [55–57], several previous works clearly state that TLR9 activated by ligand (CpG-ODN) induces neuroprotection [58,59] and stimulates the neuroprotective role of microglia [60,61] without production of neurotoxic mediators such as TNF-α [61]. The contradictory results may be attributable to the effect of CpG-ODN on different microglial phenotypes [49]; in the present study, CpG-ODN might mimic the effect of NSCs to instruct microglia towards a beneficial phenotype.

Upon activation, microglia release a large number of substances that can act detrimentally or beneficially for the surrounding cells [50]. Various cytokines, inflammatory factors and also neuronal signalling molecules have been reported to actively control microglial function, thus introducing microglia towards a detrimental or beneficial phenotype [50,62,63]. In the present study, first, we showed that NSCs co-culturing significantly increased the protein level of IBA-1 in the brain slices, primary microglia cells and BV2 microglia, which is consistent with the study that neural progenitor cells conditioned medium or injection resulted in increases in the number of IBA-1^+^ microglia [21]. Additionally, microglial activation is considered to be reflected by the morphological transformation [64]. Here, we observed that NSCs induced significantly microglial morphological transformation from a resting state ‘immotile’ to an activated state ‘locomotory’ in the brain tissue. Microglia in locomotory state is reported to actively contact with other cells or phagocyte dead cells [36]. Second, we demonstrated that NSCs reduced the expression of TNF-α in the slices, primary microglia cells or BV2 microglia, thus supporting their ability to inhibit pro-inflammatory molecules expression. Contrarily, we detected a significant up-regulation of IL-10, a cytokine found to be positively correlated with the capacity of phagocytosis, which facilitates inflammation resolution and plays a key role in promoting repair of the CNS [65]. Moreover, before co-culturing with NSCs, there was no significant difference in the mRNA expression of IBA-1, TNF-α and IL-10 between the two comparable groups, thus confirming that the effect of microglial agonist or antagonist on the neuronal survival in the brain slices may not follow the effect of cytokines.

Additionally, we demonstrated that NSCs can induce a significant up-regulation of surface molecules on microglia associated with neuroprotective phenotype. Among these, augmenting CX3CR1 signalling has been shown to decrease microglial neurotoxicity [16,66]; up-regulation of TREM2, an innate immune receptor expressed by microglia, has been shown to be involved in phagocytosis in the absence of inflammation [40]. Increasing expression of IGF-1 was considered to play a key role in promoting repair of the CNS [67]. These data indicated that NSCs especially promote the switch of microglia from a detrimental, neurotoxic phenotype dominated by the expression of pro-inflammatory molecules to a beneficial, neuroprotective phenotype associated with the production of anti-inflammatory factor, trophic factor and calming receptors.

Several molecules may be involved in this NSCs-induced microglial activation, either constitutively expressed by microglia or produced constitutively by NSCs and released following cross-talk with target tissue. Here, we addressed the molecules expressed by microglia. Toll-like receptors represent a major class of innate immune receptors abundant on the surface of microglia [68]. Among the TLRs, TLR2, TLR4 and TLR9 are critical for microglial phagocytosis; they promote phagocytosis to varying degrees with TLR9 being the strongest one [60,68,69]. Toll-like receptor-9 pathway is mainly activated by the hypomethylated CpG-DNA, which is a common feature of bacterial and virus DNA [70], but it is also present in mammalian DNA promoter elements. TLRs could also recognize endogenous ligands released from stressed mammalian cells [71]. Here, we showed that the TLR9 mRNA level was up-regulated by NSCs and was positively correlated with IBA-1 mRNA level. The result suggested that TLR9 not TLR4 or TLR2 may be involved in NSCs-induced microglial activation.

CpG-ODN has been found to promote microglial activation [56,60,61] and this was reliant on ERK1/2 signalling. It appears that microglial activation has a strict requirement of ERK signalling. In line with the findings that ERK was a key regulator of microglial activation [23–25], we observed that NSCs enhanced the phosphorylation level of ERK1/2, which was positively correlated with IBA-1 protein level in the brain slices. The results represent a mechanism that TLR9 and ERK1/2 signalling may mediate NSCs-induced host microglial activation. However, there is some controversy surrounding the specific point at whether TLR signalling initiates ERK1/2 activation or ERK1/2 activation initiates TLR signalling [72]. Some previous works clearly state that TLR9 ligand CpG-ODN induced the activation of ERK1/2 [27,28]. Consistent with these findings, our result demonstrated that chloroquine treatment abrogated the effect of NSCs or CpG-ODN on the protein expression of TLR9, IBA-1 and phosphorylated ERK1, whereas U0126 treatment decreased NSCs- or CpG-ODN-induced ERK1/2 activation and IBA-1 expression, but not the TLR9 expression, which suggests that TLR9 dependent ERK1/2 pathway may be involved in NSCs-mediated microglial phenotype changing.

As the adult brain tissue was cultured separated by a membrane (0.4 lm pore size) from the NSCs, thus preventing direct interaction (see Materials and methods section). This suggests that diffusible factors produced constitutively by NSCs or released following cross-talk with target tissue can activate TLR9-ERK pathway and switch microglia to a protective phenotype; more work focus on ‘how NSCs activate TLR9-ERK pathway’ is being arranged for our future study.

In conclusion, NSCs improved neuronal survival by switching microglia from a detrimental to a neuroprotective phenotype, and the microglial TLR9-ERK1/2 pathway seems to participate in this NSCs-mediated neurorescue. These results might provide novel therapeutic strategies on NSCs-based treatment of neurological disease.

## Supporting information

**Figure S1**. Representative images of different type of cells in neonatal and 15-month-old mouse brain slices.

**Figure S2**. Minocycline has no effect on the multipotency and undifferentiated state of NSCs.

**Figure S3**. The cytokine expression in the brains slices of the comparable group before co-culturing with or without NSCs.

**Figure S4**. NSCs affected the expression of effector molecules in BV2 cell line.

**Figure S5**. Microglia was activated *via* TLR9-ERK1/2 pathway in BV2 cells.

**Figure S6**. Quantification of different type of cells in the brain slices co-cultured with or without NSCs along with the co-culture time.

**Table S1**. Sequence of primers, gene bank accession numbers.

**Video S1**. Time-lapse confocal image of microglial morphological phenotype in live adult brain slices co-cultured with NSCs.

**Video S2**. Time-lapse confocal image of microglial morphological phenotype in live adult brain slices from the mock-treated group.

**Video S3**. The trajectory of locomotory microglia.

**Video S4**. The trajectory of motile microglia.

**Video S5**. The trajectory of immotile microglia.
