# Supplementary figures and images for: Microglial activation mediates host neuronal survival induced by neural stem cells
Source: J Cell Mol Med. 2014 Apr 13;18(7):1300–12. doi: 10.1111/jcmm.12281 (PMC4124015; doi:10.1111/jcmm.12281)

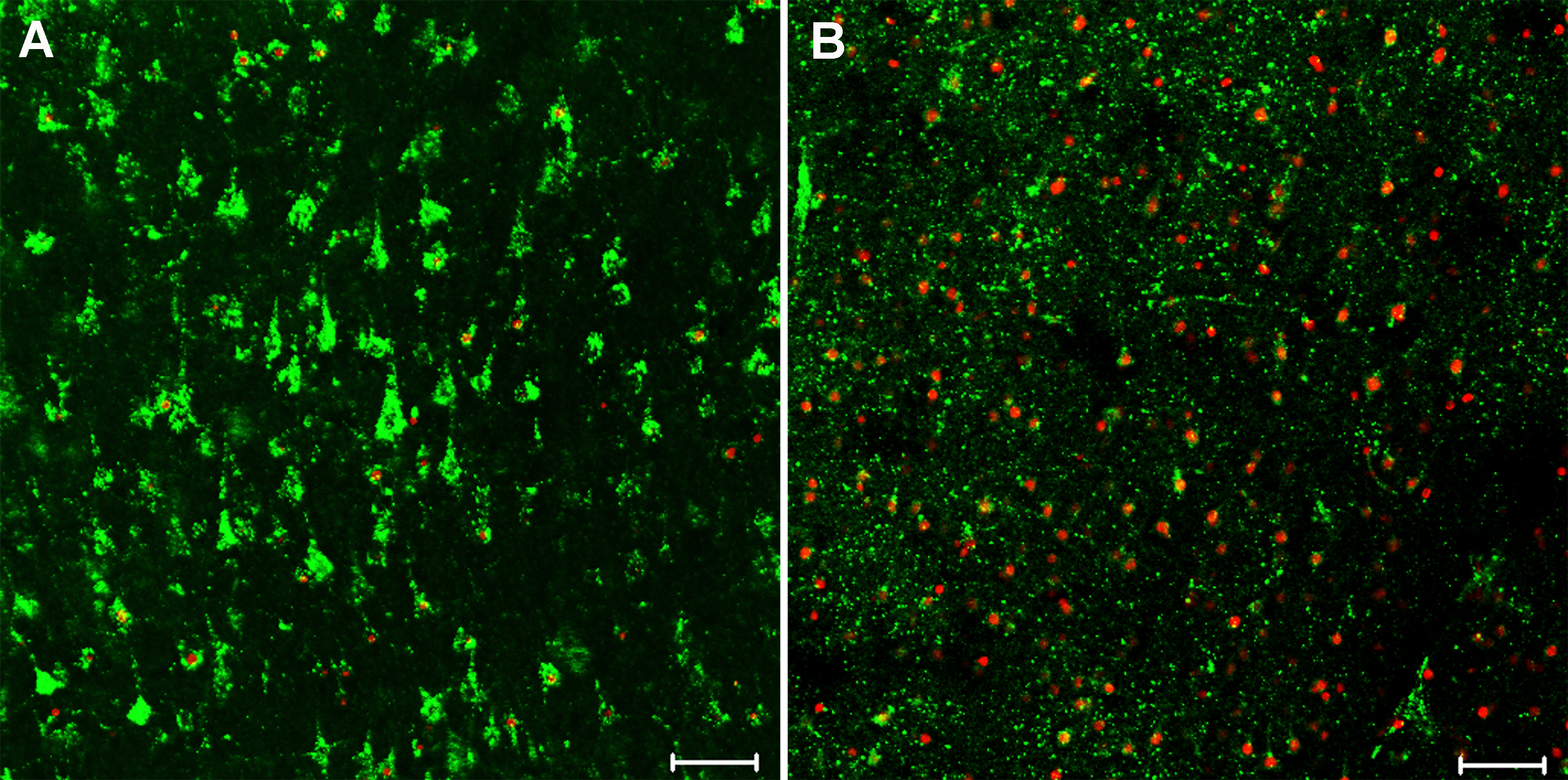

Supplement: Supplementary file 1 — Figure S1. Representative images of different type of cells in neonatal and 15-month-old mouse brain slices. [file jcmm0018-1300-SD1.tif]

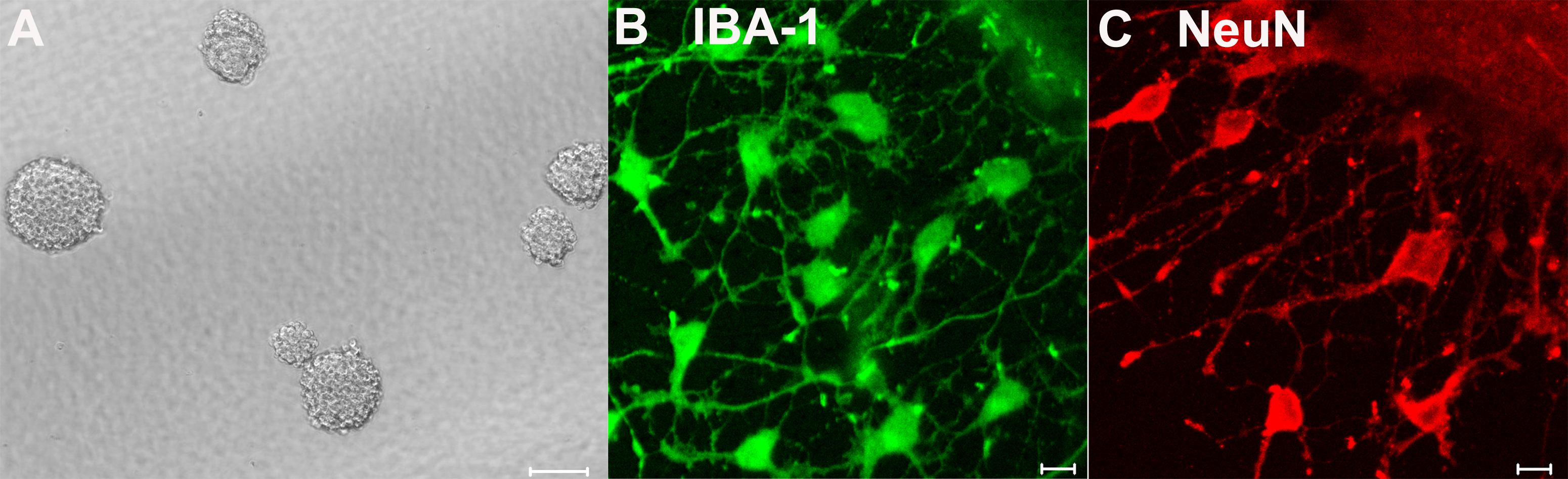

Supplement: Supplementary file 2 — Figure S2. Minocycline has no effect on the multipotency and undifferentiated state of NSCs. [file jcmm0018-1300-SD2.tif]

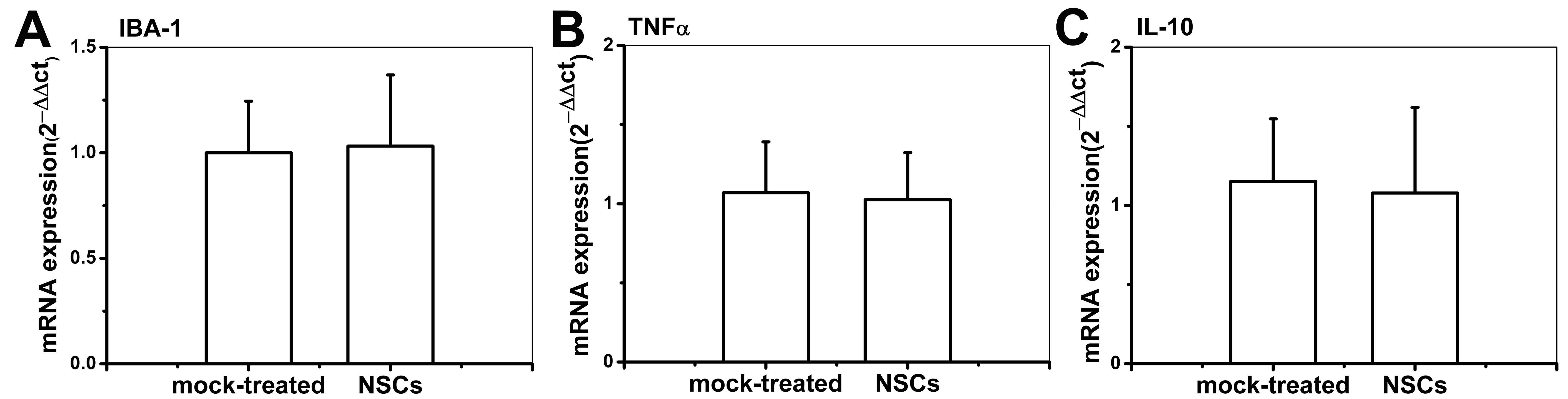

Supplement: Supplementary file 3 — Figure S3. The cytokine expression in the brains slices of the comparable group before co-culturing with or without NSCs. [file jcmm0018-1300-SD3.tif]

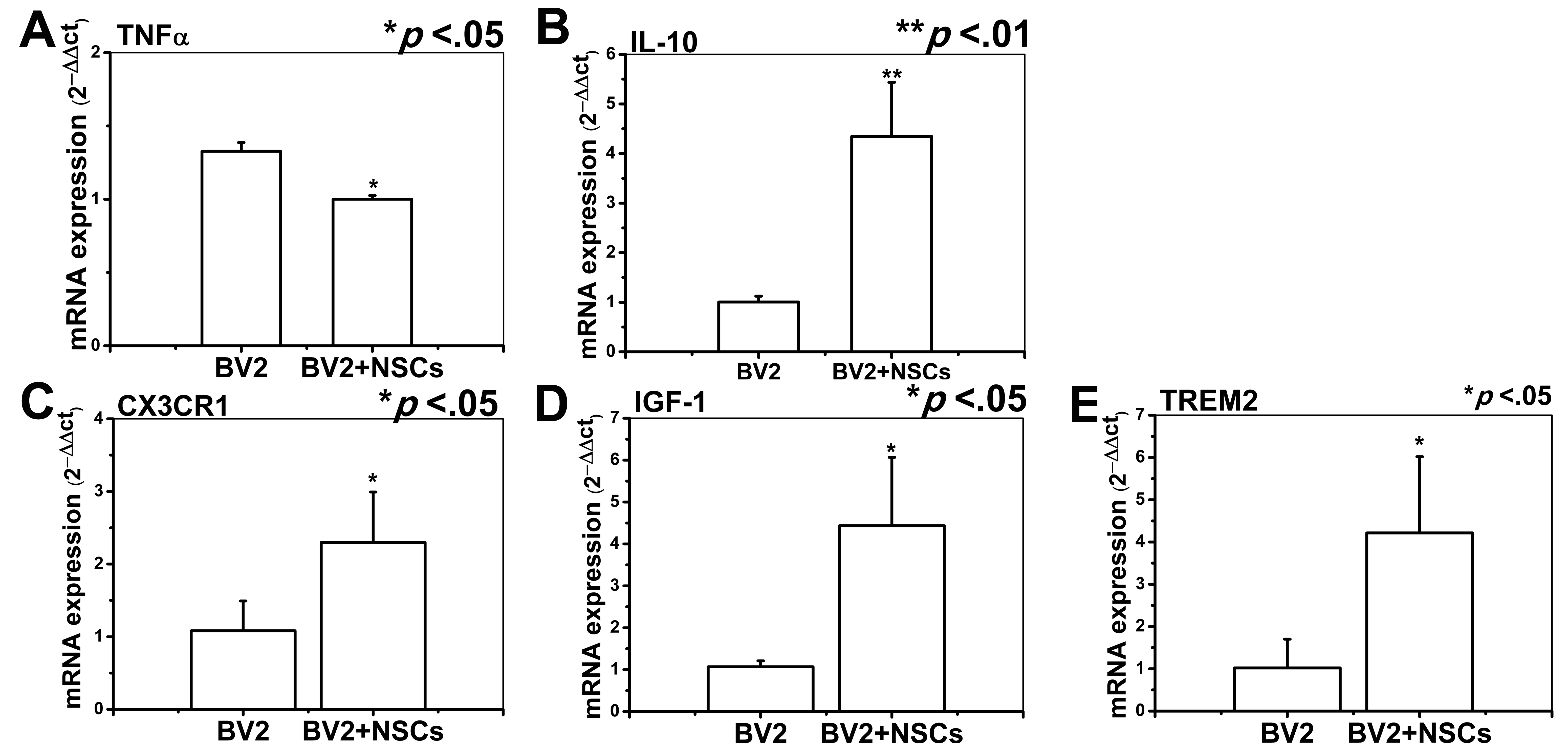

Supplement: Supplementary file 4 — Figure S4. NSCs affected the expression of effector molecules in BV2 cell line. [file jcmm0018-1300-SD4.tif]

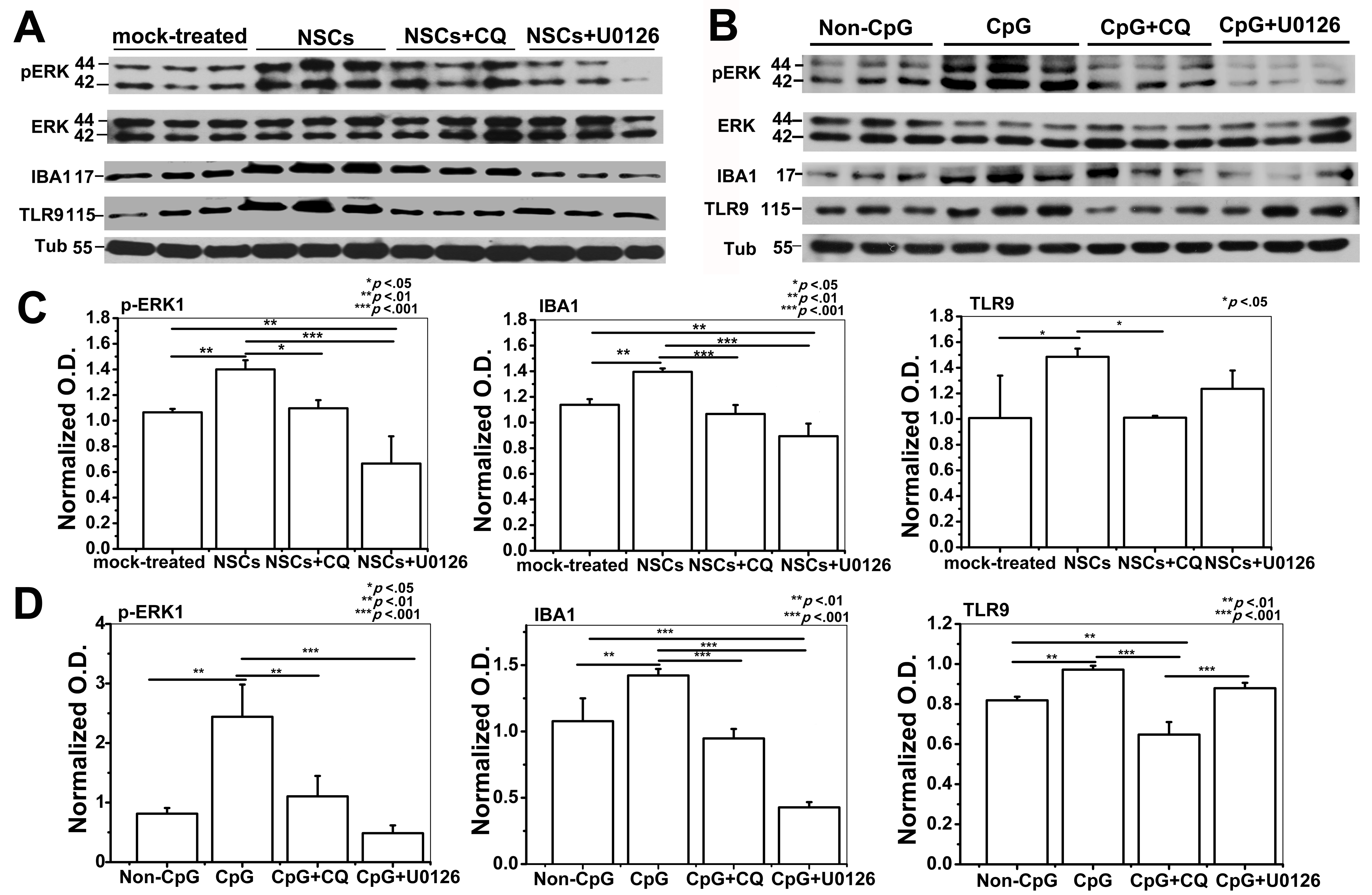

Supplement: Supplementary file 5 — Figure S5. Microglia was activated via TLR9-ERK1/2 pathway in BV2 cells. [file jcmm0018-1300-SD5.tif]

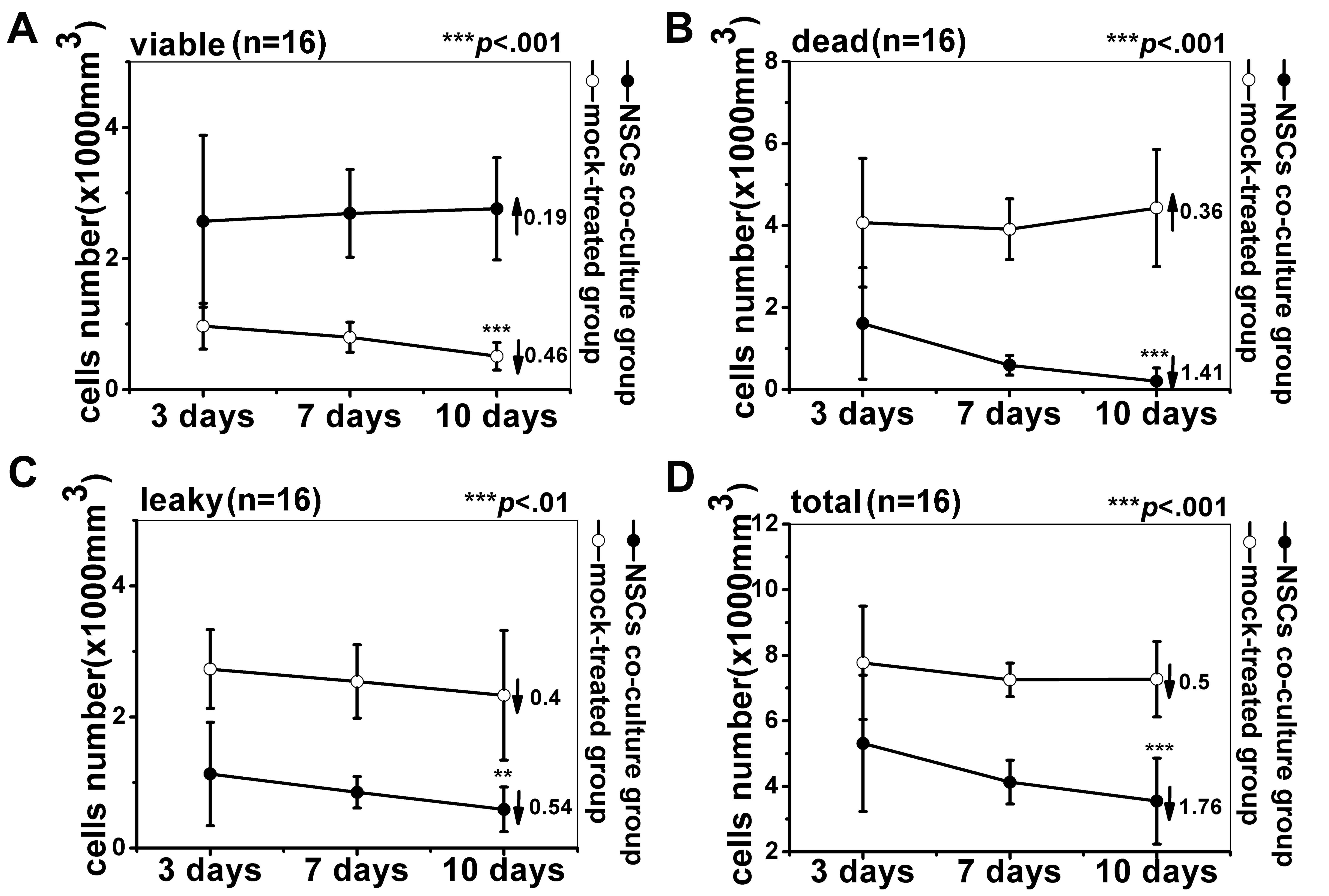

Supplement: Supplementary file 6 — Figure S6. Quantification of different type of cells in the brain slices co-cultured with or without NSCs along with the co-culture time. [file jcmm0018-1300-SD6.tif]
