# Supplementary material for: Microglial activation mediates host neuronal survival induced by neural stem cells
Source: J Cell Mol Med. 2014 Apr 13;18(7):1300–12. doi: 10.1111/jcmm.12281 (PMC4124015; doi:10.1111/jcmm.12281)
Supplement: Supplementary file 7 — Table S1. Sequence of primers, gene bank accession numbers. [file jcmm0018-1300-SD7.doc]

**Supplementary Materials:**

**Supp Fig. S1. Representative images of different type of cells in neonatal and 15-month old mouse brain slices**. After one day cultured in vitro, the slices were stained using Live/Dead kit. The cells in the neonatal brain slice survival well, however, the cells in aged mice brain slice (15-month old) die quickly. **(A)** The neonatal mouse brain slices cultured in vitro. **(B)** The 15-month old mouse brain slices cultured in vitro. Bar 50μm**.**

**Supp Fig. S2. Minocycline has no effect on the multipotency and undifferentiated state of NSCs.** After three days co-cultured with brain slice in the presence of minocycline, **(A)** NSCs remained the morphology as neurosphere, after removing EGF and FGF, and cultured with serum containing medium, many cells had differentiated towards several neural cell types (B–C). Bar: 50μm. **(B)** cells expressing microglia maker IBA-1. Bar: 10μm. **(C)** cells expressing neuron maker NeuN. Bar: 10μm.

**Supp Fig. S3. The cytokine expression in the brains slices of the comparable group before co-culturing with or without NSCs.** The expression of cytokines was analyzed by real time polymerase chain reaction. Before co-culturing with or without NSCs, we found no significant difference of microglial maker IBA-1 expression **(A)** in the slices from the two comparable groups, and also the same expression level of TNFα and IL-10 **(B-C)**. (n=5)

**Supp Fig. S4.** **NSCs affected the expression of effector molecules in BV2 cell line.** The expression of effector molecules were analyzed by real time polymerase chain reaction as described in Materials and methods. **(A-B)** NSCs affected TNFα and IL10 mRNA expression in the BV2 cells. **(C-E)** NSCs increased CX3CR1, IGF-1 and TREM2 mRNA expression in the BV2 cells. Results are shown as means ± SD of at least three independent experiments. Data were expressed as means ± SD from at least three independent experiments.

**Supp Fig. S5 Microglia was activated via TLR9-ERK1/2 pathway in BV2 cells. (A)** Western blotting analysis showed the protein expression of ERK1/2, TLR9 and IBA-1 in BV2 microglia after 3 days co-cultured with or without NSCs, or with NSCs in the presence of CQ, U0126. **(B)** Western blotting analysis showed the protein expression of ERK1/2, TLR9 and IBA-1 in BV2 microglia after 24h treated with non CpG, CpG, CpG+CQ, CpG+U0126. **(C)** Data showed the protein level of TLR9, phosphorylated ERK1 and IBA-1 in BV2 microglia co-cultured with or without NSCs, or with NSCs in the presence of CQ, U0126. **(D)** Data showed the protein level of TLR9, phosphorylated ERK1 and IBA-1 in BV2 cells treated with non-CpG, CpG, CpG+CQ, CpG+U0126. Data were expressed as means ± SD from at least three independent experiments.

**Supp Fig. S6** **Quantification of different type of cells in the brain slices co-cultured with or without NSCs along with the co-culture time.** At 3, 7 and 10 days, the slices were stained using Live/Dead kit according to the Material and methods, the cells were defined as viable cells, dead cells and leaky cells. **(A)** Number of viable cells. **(B)** Number of dead cells **(C)** Number of leaky cells. **(D)** Number of total cells. Results were shown as means ± SD (n=16).

**Supp Table S1.** Sequence of primers, gene bank accession numbers

| Gene | Left | Right |
| --- | --- | --- |
| TLR9 | ATG GTT CTC CGT CGA AGG ACT | GAG GCT TCA GCT CAC AGG G |
| TLR4 | CCT GAT GAC ATT CCT TCT | AGC CAC CAG ATT CTC TAA |
| TLR2 | TCT CTG GAG CAT CCG AAT TG | CCT GAG CAG AAC AGC GTT TG |
| IBA-1 | GGA TTT GCA GGG AGG AAA AG | TGG GAT CAT CGA GGA ATT G |
| TNFα | TCC CAG GTT CTC TTC AAG GGA | GGT GAG GAG CAC GTA GTC GG |
| IL10 | ATG CTG CCT GCT CTT ACT GAC TG | CCC AAG TAA CCC TTA AAG TCC TGC |
| CX3CR1 | CGT GAG ACT GGG TGA GTG AC | CTC ACC ATG TCC ACC TCC TT |
| TREM2 | GCA CCT CCA GGA ATC AAG AG | GGG TCC AGT GAG GAT CTG AA |
| IGF-1 | CTG GAC CAG AGA CCC TTT GC | AGA GCG GGC TGC TTT TGT AG |
| β-actin | TGT TAC CAA CTG GGA CGA CA | GGG GTG TTG AAG GTC TCA AA |

Abbreviations: TLR9: toll like receptor 9; TLR4: toll like receptor 4; TLR2: toll like receptor 2; TREM2: triggering receptor expressed on myeloid cells 2; IBA-1: ionied calcium binding adaptor molecule 1; CX3CR1: CX3C chemokine receptor 1; IGF-1: insulin growth factor 1; TNFα: tumor necrosis factor α; IL10: interleukin 10.

**Supp Movie S1.** Time-lapse confocal image of microglial morphological phenotype in live adult brain slices co-cultured with NSCs

**Supp Movie S2.** Time-lapse confocal image of microglial morphological phenotype in live adult brain slices from the mock-treated group.

**Supp Movie S3.** The trajectory of locomotory microglia

**Supp Movie S4.** The trajectory of motile microglia

**Supp Movie S5.** The trajectory of immotile microglia
